# 432. An Outbreak of Coronavirus Disease, 2019 (COVID-19) in a Skilled Nursing Facility – California, 2021: Description, Mitigation, Challenges, and Opportunities

**DOI:** 10.1093/ofid/ofab466.632

**Published:** 2021-12-04

**Authors:** Anastasia Maletz, Grace Kang, Raymond Y Chinn, John D Malone, Hosniyeh Bagheri, Margaret M Turner, Elizar Perez, Michelle Hose, Seema Shah, Mark Zeller, Kristian Anderson, Eric McDonald, Jacqueline Ruegg, Sandra Brackman

**Affiliations:** 1 UC San Diego Health, San Diego, California; 2 County of San Diego, Epidemiology & Immunization Services Branch, San Diego, California; 3 County of San Diego, Health and Human Services Agency, San Diego, California; 4 California Department of Public Health (CDPH), Rancho Santa Margarita, CA; 5 California Department of Public Health, Santee, California; 6 California Department of Public Health, Center for Healthcare Quality, San Diego, California; 7 The Scripps Research Institute, San Diago, California; 8 County of San Diego, San Diego, CA; 9 SD County Public Health Department, Ramona, California

## Abstract

**Background:**

Skilled nursing facility (SNF) residents comprised 11% of all COVID-19 cases in the United States; however, they account for 43% of deaths with case fatality rates (CFR) of 26.0-33.7%.

**Methods:**

We report an outbreak of COVID-19, from June 15 to July 21, 2020 in a 159-bed SNF with a staff of 172 that resulted in an infection rate of 97% in residents and 23% in HCWs (Figure 1). A retroactive review outlined mitigation efforts, discussed challenges, identified risk factors among residents and health care workers (HCW) for acquisition of COVID-19, and reviewed opportunities for improvement (Figure 2).

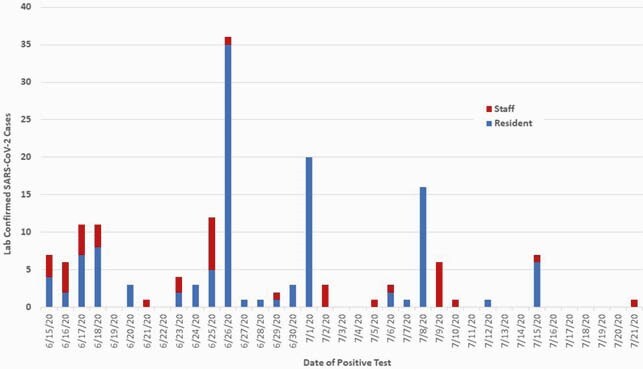

Figure 1. Epi Curve of COVID-19 Outbreak in a Skilled Nursing Facility

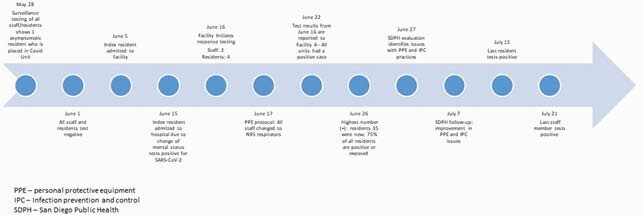

Figure 2. Timeline of COVID-19 Outbreak in a Skilled Nursing Facility

**Results:**

Factors that contributed to the outbreak: delay in test results had an impact on cohorting; suboptimal adherence to the principles of infection prevention and control (IPC) and minimal adherence monitoring; strict criteria were used to screen for infection; the underappreciated transmissibility of COVID-19 from presymptomatic and asymptomatic persons; symptomatic HCWs who continued to work; the changing guidance on, the suboptimal use of, and an inadequate supply of personal protective equipment; poor indoor air quality due to ventilation challenges; and the important role of community/family/interfacility spread on the outbreak. Whole genome sequencing, performed in 52 samples, identified a common strain that was also found in clusters of 2 other facilities: 1 in the same geographic location, the other in a different geographic location but whose HCWs had the same zip codes as the facility (Figure 3). Certified nursing and restorative nursing assistants had the highest risk of infection with an odds ratio (OR) of 4.02 (confidence interval 1.29-12.55, p value: 0.02) when compared to registered and licensed vocational nurses. The residents’ CFR was 24%. The OR for death was increased by 10.5 (10.20-11.00) for every decade of life as was morbid obesity (BMI > 35) with an OR of 8.50. BMI as a continuous variable increased risk of mortality for every additional unit, OR 1.07 (Tables 1, 2).

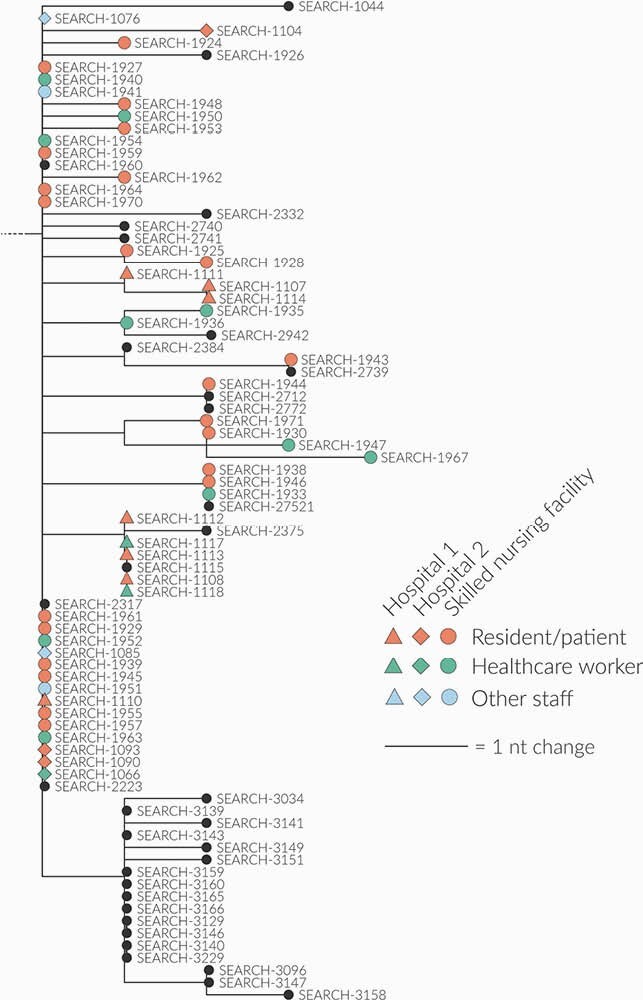

Whole Genome Sequencing of Isolates from a Skilled Nursing Facility Outbreak

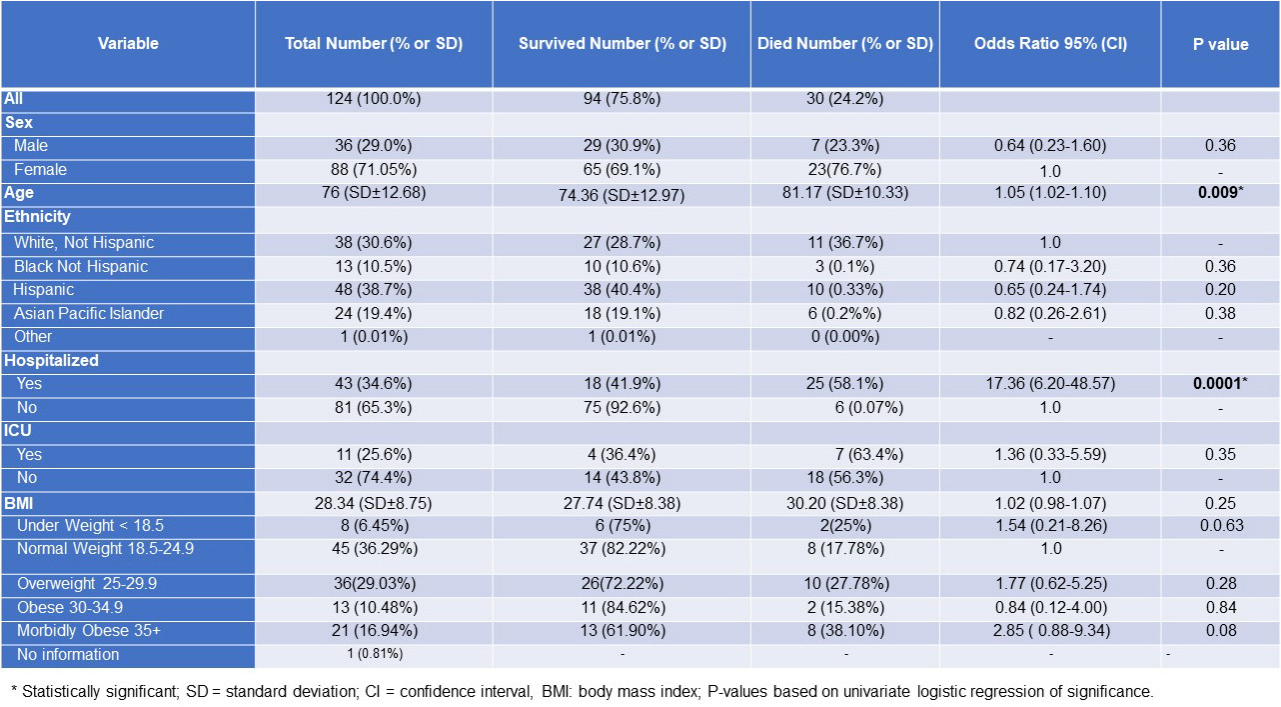

Univariate Analysis of Selected Variables Associated with Mortality among Residents at Facility A during COVID-19 Outbreak, June 19 - July 21, 2021

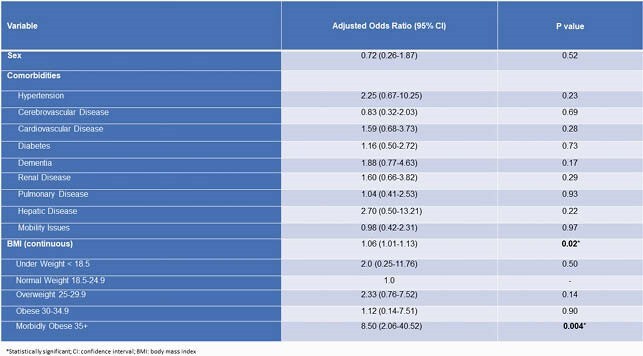

Multivariate Analysis of Factors Associated with Mortality from COVID-19 after Adjusting for Age among Residents (N =124) of Facility A, June 15 - July 21, 2020

**Conclusion:**

While implementation of optimal IPC measures in the pre-COVID-19 vaccination era had no impact on the infections in residents who were likely already infected or exposed at the onset of the outbreak, these measures along with non-pharmacologic strategies were effective in halting the spread among HCWs.

**Disclosures:**

**All Authors**: No reported disclosures

